# Impact of front-of-pack nutrition labels on consumer purchasing intentions: a randomized experiment in low- and middle-income Mexican adults

**DOI:** 10.1186/s12889-020-08549-0

**Published:** 2020-04-06

**Authors:** Alejandra Jáuregui, Jorge Vargas-Meza, Claudia Nieto, Alejandra Contreras-Manzano, Nelson Zacarías Alejandro, Lizbeth Tolentino-Mayo, Marissa G. Hall, Simón Barquera

**Affiliations:** 1 Nutrition and Health Research Center, Mexican National Institute of Public Health, Av. Universidad 655 Col. Santa María Ahuacatitlán, CP. 62100 Cuernavaca, Morelos Mexico; 2 School of Public Health, Mexican National Institute of Public Health, Av. Universidad 655 Col. Santa María Ahuacatitlán, CP. 62100 Cuernavaca, Mexico; 3 Department of Health Behavior, Gillings School of Global Public Health, University of North Carolina and Lineberger Comprehensive Cancer Center, University of North Carolina, Chapel Hill, USA

**Keywords:** Nutritional labeling, Latin-America, Shopping time

## Abstract

**Background:**

Front-of-pack (FOP) nutrition labeling is a cost-effective strategy to help consumers make informed and healthier food choices. We aimed to investigate the effect of the FOP labels used in the Latin American region on consumers’ shopping intentions when prompted to make their choices with specific nutrients-to-limit in mind among low- and middle-income Mexican adults (> 18 y).

**Methods:**

In this experimental study of an online simulated shopping situation participants (*n* = 2194) were randomly assigned to one of three labeling conditions: Guideline Daily Amounts (GDA), Multiple Traffic Lights (MTL), or red Warning Labels (WL). Participants were required to view a video explaining how to correctly interpret the assigned label. Primary outcomes were the overall nutritional quality (estimated using the Nutrient Profiling Scoring Criterion [NPSC] and NPSC baseline score) and mean energy and nutrient content of purchases. Secondary outcomes included shopping time variables. We also evaluated the impact of the labels across food categories (ready-made foods, dairy beverages, non-dairy beverages, salty snacks, and breakfast cereals) and sociodemographic subgroups.

**Results:**

The MTL and the WL led to a better overall nutritional quality of the shopping cart compared to the GDA (*p* < 0.05). According to the NPSC score, the WL led to a better nutritional quality across breakfast cereals and salty snacks compared to the GDA (*p* < 0.05); a similar effect was observed for the MTL among non-dairy beverages (*p* < 0.05). The MTL and the WL required shorter shopping times compared to GDA (*p* < 0.05). Across all labeling conditions, the nutritional quality of the shopping cart tended to be lower among those with low income, education and nutrition knowledge levels.

**Conclusion:**

WL and MTL may foster healthier food choices in a faster way among low- and middle-income groups in Mexico. To produce an equitable impact among consumers of all socioeconomic strata, efforts beyond simply the inclusion of a communication campaign on how to use and interpret FOP labels will be required.

**Trial registration:**

clinicaltrials.gov. NCT04308408 Retrospectively registered March 16, 2020.

## Background

Front-of-pack (FOP) nutrition labeling is part of the emerging structural actions to improve the food environment to address the growing global burden of diet related noncommunicable diseases [1]. It is a cost-effective strategy to help consumers make healthier food choices [2]. Several countries globally have implemented a mandatory FOP nutrition label on processed foods [3], and discussions are being held at the highest international levels, including the Codex Alimentarius Commission, the internationally recognized standards-setting body [4].

According to Grunert and Wills [5], for nutrition labels to have any effect on purchasing decisions, consumers must first be exposed to them and perceive the displayed information on the labels. Then, the effect will be mediated by consumer understanding. Based on this understanding, consumers may use the label to make inferences about the nutritional quality or healthiness of the product, which, together with other information (e.g. trust or liking of the label or taste of the product) may affect the evaluation of the product and eventually the purchase decision of the product.

Recently, a variety of FOP labels have been adopted in Latin America [3]. Traffic light labels, an interpretive nutrient-specific FOP label, were implemented as a mandatory regulation in Ecuador in 2013. This label uses the typical traffic light colors (green, yellow/amber, red) and text descriptors to indicate the high, medium, or low content of total fat, sugar and salt. In 2016, Chile introduced Warning Labels (WL), another nutrient-specific interpretive FOP labelling scheme, requiring ‘high in’ symbols for products that exceed limits of energy, sodium, sugar and saturated fat. Soon afterwards Uruguay and Peru implemented similar warning labels as their mandatory FOP labeling scheme, and Brazil is in the process of developing a similar system.

In contrast with these two interpretive labeling approaches, Guideline Daily Amounts (GDA) is a purely numerical and reductive labeling system indicating the grams and percentages (according to the guideline-based daily intakes) per portion of kilocalories, saturated fats, other fats, sugars, and sodium, with no specific judgement, opinion or recommendation. In 2011, GDA’s were implemented by the food industry in Mexico as a voluntary label, along with more than 5 million USD invested in national communication and educational campaigns [6]. In 2016, GDA’s were adopted as the mandatory FOP label, despite evidence indicating this was not the best FOPL approach for the Mexican population [7]. Consistent evidence indicates that GDA are not understood or used by the Mexican population [8–12]. Therefore, Mexican authorities are considering modifications to current labeling regulations to replace GDA’s with a more effective labeling approach.

An increasingly large number of studies evaluating FOP nutrition labels’ impact on awareness [13, 14], acceptability [15], understanding [14, 16, 17], use [13], or effects in healthfulness perceptions [18, 19], have been conducted among Latin American populations. However, limited literature exists evaluating FOP label effects on consumer purchasing intentions (e.g. through online shopping simulations) or actual purchases (e.g. using purchase data) among this population [20–23], with some studies indicating that FOP labels may improve the nutritional quality of consumers’ choices [22, 23], and others reporting limited or null effects [20, 21]. Evidence suggests that nutritional labels may be more effective when supported by measures to promote awareness, understanding and use of such labels [24, 25], specifically when considering health-related outcomes [26]. Additionally, some studies have included highly educated participants [11, 27], limiting the representativeness of results among lower income and education groups, which are the most representative of the Latin-American populations and generally the most nutritionally at-risk [28].

To address these gaps in literature, we aimed to assess the effect of three FOP nutrition labels on consumers’ food choice when prompted to make their choices with specific nutrients in mind in low- and middle-income Mexican adults using a randomized experiment of an online simulated shopping situation. The present study could provide evidence to support policies aiming to implement effective FOP labeling systems at the national and regional level and generate academic and political discourse globally.

## Methods

### Study design

**Fig. 1:**
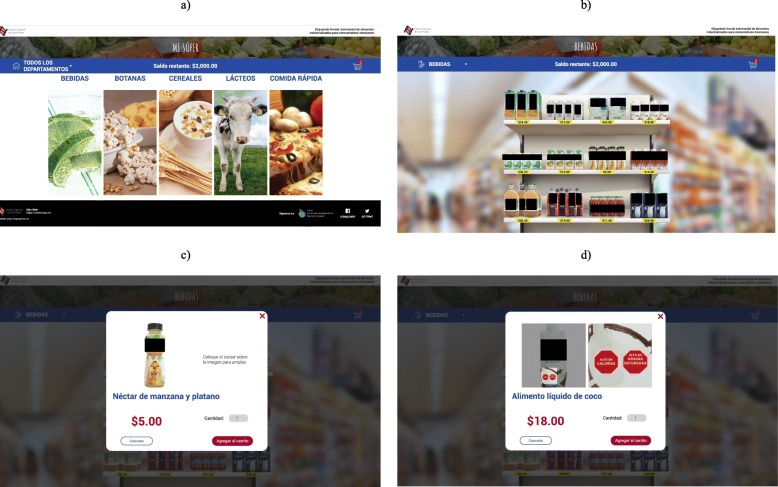
The online shopping site. **a** Food categories. **b** Products on shopping shelves; **c** Pop-up window displaying product information; **d** Zoom into the package and the nutritional label. Source: Figure prepared by authors using the website purposively created by the research team for this experiment

A three-arm unblinded randomized experiment was conducted from May to June 2018. This was a two-part study exploring the effects of different FOP labels among a sample of Mexican consumers. The first part consisted on a randomized experiment of an online shopping simulation. The second part used a randomized experiment to explore the understanding of the labels [12]. This paper reports the results of the first part of the study. The online shopping simulation employed an online grocery store developed for this purpose to simulate a shopping situation (Fig. 1). The ethics, research and biosafety committees of the Mexican National Institute of Public Health evaluated and approved this study (approval number: 1122).

### Recruitment

The methods for this experimental study have been reported previously [12]. Briefly, trained undergraduate student research assistants (ages 18 and above) from eight universities across the country recruited the study participants. Research assistants were trained on how to approach and recruit participants and obtain informed consent. They were instructed to recruit 20 or more participants each. Recruitment took place in public spaces previously selected by convenience by our research team, based on their use by low- and middle-income groups in Mexico (i.e., public schools, public squares, public health centers, as well as supermarket chains and shopping centers located in low-income neighborhoods). Potential participants were approached by research assistants, who explained study objectives and invited them to be part of the study. Then, individuals were screened for eligibility using a 3-item screener. To access this screener, research assistants used a tablet or laptop with internet-access, to access a unique web address where our web-based tool was hosted. Eligible participants were adults (> 18 years old) consuming at least one of the five food groups included in the experiment (see below) and involved in grocery shopping at least twice per week. Participants were excluded if they or any of their direct relatives worked for the food or beverage industry. Research assistants were automatically informed by the web-based tool about the eligibility of the participant. Informed consent was obtained from all eligible participants using an automated computer-based form. Then, the tablet or laptop was handed to participants, who completed a self-administered online demographic and health survey, and then accessed the online shopping site to simulate a shopping situation. Participants completed the shopping task at the public place where they were recruited.

### Participant’s allocation

**Fig. 2:**
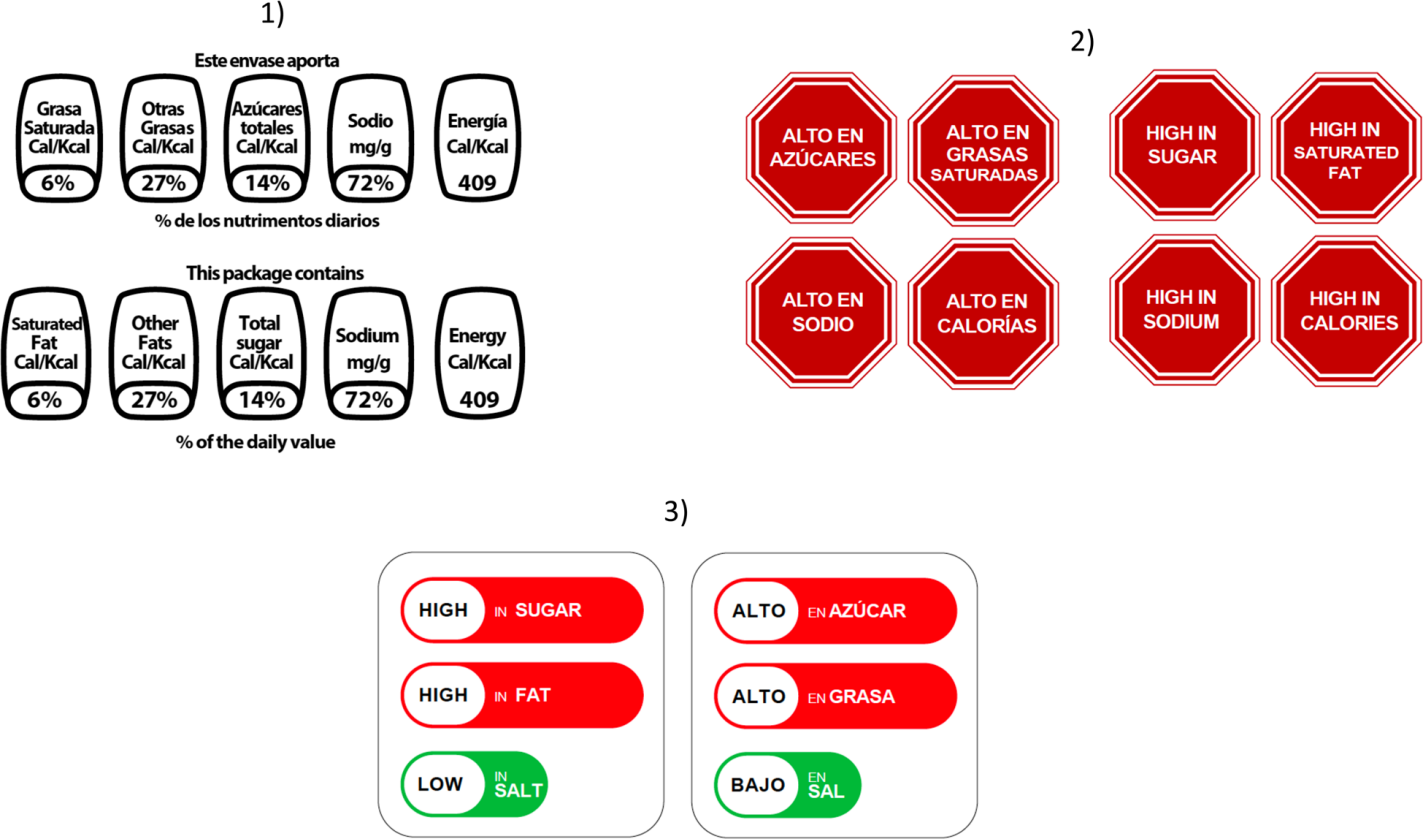
Front-of-pack labeling systems tested. 1) Guideline Daily Allowances, 2) Multiple Traffic Lights used in Ecuador, 3) Red Warning Labels similar to Chile’s labeling system

Participants were randomly assigned to one of three FOP labels using a central computer system (Fig. 2), blinding the research assistants to the assigned condition. Blinding of participants was not possible given the nature of the intervention. FOPL included the following:

1. Mexican GDA. The GDA label was the control group because they are the mandatory front of pack labelling system in Mexico.
2. Ecuadorian Multiple Traffic Lights (MTL).
3. Chilean Warning Labels (WL) in red. Red WL were used because previous work in Mexican consumers has demonstrated this color increased label acceptability [29] and discouragement from wanting to consume unhealthy drinks [30], both of which are predictors of the effectiveness of FOP labels [5, 25, 31]

### Intervention

After allocation, participants viewed a video explaining how to correctly interpret the assigned label (the prompt to make their choices with specific nutrients-to-limit in mind). We used official videos used in each country (i.e. Mexico, Ecuador and Chile) to promote the correct use of the current labeling. We chose to show participants videos to mimic the real-world implementation of FOP labeling policies in which the policy roll-out is often accompanied by an educational media campaign. Participants in the GDA condition viewed a video from Mexico, corresponding to the one prepared in 2011 by a food industry coalition and disseminated in the following years as part of the educational campaign to promote the use of GDAs. It consists of an explanation on how to interpret GDAs using animations and text and encouraging consumers to “Check and Choose” wisely. Participants in the MTL arm viewed a video prepared by the Ministry of Public Health and the Ministry of Human Development Coordination of Ecuador in 2014. The video shows a child with a food cart while shopping at a supermarket. The video highlights a variety of chronic diseases (e.g., diabetes, cardiovascular disease) and its key message is to “choose right to live right”. Finally, participants in the WL condition viewed a video prepared by the Ministry of Health of Chile and shows a variety of population groups (adults, children, adolescents and older adults) in different contexts (shopping at a supermarket, cooking at home, playing outdoors or eating at school). It highlights childhood obesity and other diseases and the main message of the video is to “Prefer foods with fewer labels, and it’s better if they don’t have any”. All videos lasted less than 1 min. Additional details on the videos are provided in Supplementary Table 1.

Each participant was asked to shop freely, to better mimic real-world grocery shopping conditions. Research assistants instructed participants as follows: “*take into account the assigned label and choose your preferred products*”. Participants were not reminded to make their choices with specific nutrients-to-limit in mind in this instruction. Participants were assigned an initial budget to do their shopping, although they did not pay actual money for their groceries. This budget corresponded to their weekly expenditure in groceries reported in the demographic survey, from 500 to 5000 MXN pesos (≈28–280 USD), in multiples of 500. No specific instructions were given related to the number or total cost of food products purchased.

FOP nutrition labels were affixed on the food items of the virtual supermarket (Fig. 1). The store displayed the name, price and the front of the pack image of 60 food products from 5 food groups (ready-made foods, dairy products, non-dairy beverages, salty snacks, and breakfast cereals) (Fig. 1a and b). These food groups were selected because they include products that have been associated with increasing diet-related non-communicable diseases (i.e., savory snacks, breakfast cereals, sweetened beverages, fast food, milk and dairy sweetened products) [32] and are susceptible to consumer-behavior change according to previous studies [33]. Food products were classified according to their nutritional quality with the Nutrient Profiling Scoring Criterion (NPSC) model and a variety of products ranging in their nutritional quality were selected. The NPSC scores the nutritional quality of food products by calculating a baseline score based on their content of “negative” nutrients (i.e. energy, saturated fat, total sugar and sodium); then, a “positive” score is calculated based on the content of desirable nutrients (i.e., protein, fiber and fruit and vegetable), which is subtracted to baseline points to estimate the final score. A negative NPSC score indicates high nutritional quality, whereas a positive score indicates a lower nutritional quality [34]. We used a database collected by the Mexican National Institute of Public Health between 2015 and 2016 to retrieve nutrient content and price information [35]. Supplementary Table 2 presents the nutritional quality and nutritional content of the foods used in the virtual supermarket. Labels were placed in the lower left corner of the front of the package (Fig. 1d). All food items in the GDA and MTL groups displayed the corresponding label; food items in the WL group only displayed a WL if the product exceeded the limit for a specific nutrient. In total, in the WL condition, 37 of the 60 food products (11 ready-made foods, 10 dairy products, 7 non-dairy beverages, 4 salty snacks and 5 breakfast cereals) did not display a WL.

Products were shown on traditional shopping shelves (Fig. 1b). Participants could zoom in to look more closely at the products and their prices, and could click on the product to access a new pop-up window (Fig. 1c). This new window displayed product information (i.e., name and brand), a larger image of the front of the product packaging, and an area where they could select the amount or number of products they wanted to put in their shopping cart. The pop-up window was the only way in which participants could select the food products to “purchase”. Participants could also zoom in further on this pop-up to look more closely at the package and the nutritional label (see Fig. 1d).

### Nutritional criteria for labeling systems

We used the nutrition criteria established by each of the FOP labels included in our study to assign the labels to food products [23, 36, 37]. Nonetheless, we used the 2016 Chilean regulations to classify nutrient content as high in the MTL and the WL [37].

### Outcomes

#### Primary outcomes

The primary outcomes of this study were the mean nutritional quality and the mean nutrient content of the shopping cart. We used the NPSC model as our main measure to evaluate the nutritional quality of the shopping cart. However, given that this model also evaluates “positive” nutrients, which are not considered in the GDA, the MTL or the WL, we also evaluated NSPC baseline score to explore a nutritional quality measure consistent with the key nutrients evaluated by the FOP labels tested. Using these two measures we calculated the overall nutritional quality of the products in the shopping cart, as well as the nutritional quality within food group categories.

The content per 100 g or 100 mL of critical nutrients (i.e., energy, proteins, total fat, saturated fat, total sugars, sodium and fiber) of the purchased items was calculated by dividing the total content of each nutrient in the shopping cart by the number of products purchased.

#### Secondary outcomes

The following variables related to the time participants spent shopping were automatically recorded: 1) time to first product selection (minutes), 2) time spent looking at a product before deciding to buy it (seconds), estimated as the time between observing the food product on the pop-up window and clicking on the “add to shopping cart” button, 3) time spent looking at a product before deciding not to buy it (seconds), estimated as the time between observing the food product on the pop-up window and going back to the shopping shelf, and 4) total time spent shopping (minutes).

We also estimated the number of products and the mean price (estimated by dividing the total cost of the shopping cart by the number of products purchased) of the products in the shopping cart, the number of times the participant viewed the product before making a purchasing decision (estimated by the number of times the participant zoomed into the product on the pop-up window), and the proportion of participants viewing the front of the pack at least once.

### Covariates

Demographics (gender, age, educational level, occupation), health information (body mass index (BMI) - estimated with self-reported height and weight, presence of chronic conditions) and knowledge about nutrition were obtained.

### Data analysis

Based on previous studies, [38] and considering a significance level of 0.05 and a power of 80%, we estimated that a total of 620 participants were needed in each label group to detect a difference of 0.2 NPSC points in the nutritional quality of the shopping cart.

We compared demographic and health characteristics of participants between experimental groups using Chi-squared tests (for categorical variables) and linear regression models (for continuous variables). Since no differences were observed between groups, analyses did not control for these variables. Means and standard deviations are presented to describe our primary outcomes. Medians and interquantile ranges were estimated for skewed variables (i.e., time variables and the number and mean price of the products in the shopping cart). The mean price of the products in the shopping cart was skewed to the right, probably because this was the distribution of the price of the products included in the online store.

To evaluate the impact of the labels on primary study outcomes, we used one-way ANOVA tests introducing the experimental group as the independent variable. Pairwise comparisons among label groups were made using Bonferroni’s multiple comparisons tests. To put the effects into a common metric, we calculated a standardized effect size (Cohen’s d) with 95% confidence intervals [39]. To test if the effect of the labels on primary outcomes differed across demographic indicators (i.e., household monthly income, education level, interest in health, and nutrition knowledge) we used linear regression models introducing an interaction term between the experimental group and the characteristic of interest (e.g., experimental group x education level).

To evaluate the impact of labels on secondary outcomes we used quantile regression models for skewed variables (i.e. time variables, number and price of the products), and one-way ANOVA tests for the number of times the participant viewed the front of the package before making a purchasing decision introducing the experimental group as the independent variable. We also tested differences in the proportion of participants viewing the front of the pack at least once across label groups using Chi squared tests. We did not have specific predictions about the impact of the labels on these secondary outcomes, so these analyses were exploratory in nature. All tests of significance were two-sided, and a *p*-value < 0.05 was considered significant. Data analysis was performed using STATA 14.

## Results

A total of 2194 participants were recruited, completed the online shopping simulation and were included in the analyses (GDA = 725, MTL = 734, WL = 735). No differences were observed in demographic and health characteristics between label groups (*p* > 0.05) (Table 1). Participants were 36.8 (±16.0) years old on average. About half of the sample (49.8%) had a monthly income below $6800 MXN (≈$357 USD), which is similar to the average income of the fifth decile of socioeconomic status in Mexico, [40] and most (51.2%) had high school education or lower. Almost half of participants (52.3%) were either overweight or obese by self-report, 78.4% reported being very interested in their health and 79.4% reported a little or some nutrition knowledge.

**Table 1:** Socio-demographic characteristics of participants by allocated front-of-pack nutrition label (*n* = 2194)

	GDA (***n*** = 725)	MTL (***n*** = 734)	WL (***n*** = 735)	Chi-square ***P*** value
**Gender, n(%)**				
Female	422 (58.2)	434 (59.1)	406 (55.2)	0.29
Male	303 (41.8)	300 (40.9)	329 (44.8)	
**Age, n(%)**				
21–29 y	348 (48.0)	350 (47.7)	363 (49.4)	0.85
30–49 y	194 (26.8)	193 (26.3)	179 (24.4)	
50 and over	183 (25.2)	191 (26.0)	193 (26.3)	
**Marital status, n(%)**				
Divorced/Single/Widow	371 (51.17)	362 (49.32)	382 (51.97)	0.58
Married/partner	354 (48.83)	372 (50.68)	353 (48.03)	
**Household monthly income (MXN pesos), mean (SD)**				
< $2699	134 (18.5)	128 (17.4)	137 (18.6)	0.99
$2700-6799	225 (31.0)	236 (32.2)	233 (31.7)	
$6800-11,599	190 (26.2)	193 (26.3)	188 (25.6)	
$11,600-34,99	126 (17.4)	125 (17.0)	133 (18.1)	
> $35,000	50 (6.9)	52 (7.1)	44 (6.0)	
**Education level, n (%)**				
Secondary school and lower	139 (19.2)	135 (18.4)	132 (18.0)	0.98
High-school	227 (31.3)	243 (33.1)	247 (33.6)	
Undergraduate	316 (43.6)	315 (42.9)	315 (42.9)	
Graduate	43 (5.9)	41 (5.6)	41 (5.6)	
**Occupation, n (%)**				
Student	184 (25.4)	185 (25.2)	176 (24.0)	0.60
Home maker	110 (15.2)	108 (14.7)	105 (14.3)	
Employee	321 (44.3)	326 (44.4)	321 (43.7)	
Salesman/woman	63 (8.7)	59 (8.0)	60 (8.2)	
Other	47 (6.5)	56 (7.6)	73 (9.9)	
**Body Mass Index categories, n (%)**^**a**^				
Underweight	15 (2.1)	15 (2.0)	23 (3.1)	0.86
Normal weight	330 (45.5)	333 (45.4)	330 (44.9)	
Overweight	287 (39.6)	292 (39.8)	293 (39.9)	
Obesity	93 (12.8)	94 (12.8)	89 (12.1)	
**Previous diagnosis of chronic disease, n (%)**				
Diabetes	57 (7.9)	62 (8.5)	63 (8.6)	0.87
Hypertension	90 (12.4)	87 (11.9)	90 (12.2)	0.95
High cholesterol	81 (11.2)	81 (11.0)	82 (11.2)	1.00
High triglycerides	70 (9.7)	77 (10.5)	85 (11.6)	0.49
**Interest in health, n (%)**^**b**^				
Not interested or a little interested	148 (20.4)	149 (20.3)	176 (24.0)	0.41
Very interested	577 (79.6)	585 (79.7)	559 (76.1)	
**Nutrition knowledge, n (%)**^**c**^				
Not knowledgeable	149 (20.5)	153 (20.8)	151 (20.5)	1.00
A little knowledgeable	299 (41.2)	300 (40.9)	306 (41.6)	
Somewhat and very knowledgeable	277 (38.2)	281 (38.3)	278 (37.8)	
**Weekly expenditure in groceries**				
Less than 1500 MXN	471 (65.0)	473 (64.4)	485 (66.0)	0.878
Between 1500 and 3000 MXN	235 (32.4)	237 (32.3)	226 (30.8)	
More than 3000 MXN	19 (2.6)	24 (3.3)	24 (3.3)	

*GDA* Guideline Daily Amount; *MTL* Multiple Traffic Light; *WL* Warning Labels

^a^ Estimated with self-reported height and weight

^b^ Information collected with the question: “*How interested are you in your health”*

^c^ Data collected with the question: *“In your opinion, how knowledgeable are you in nutrition?”*

### Nutritional quality of the shopping cart

According to the NPSC score and NPSC baseline score, the MTL and the WL led to a better overall nutritional quality of the shopping cart compared to the GDA (all *p* < 0.05) (Table 2). The MTL led to an overall NPSC score of 0.8 compared to 1.3 in GDA group (*d* = −.17, 95% CI: −.27 to −.07). The WL led to an overall NPSC score of 0.9, reflecting better quality than the 1.3 score for the GDA group (*d* = −.14, 95% CI = -.03 to −.24). Examining the NSPC base points showed a similar pattern, with the MTL leading to 6.2 base points, compared to 6.7 in the GDA group (d = −.17, 95% CI = -.27 to −.17). The WL also led to better quality NSPC base points (6.1) than the GDA group (d = −.20, 95% CI = -.30 to −.10).

**Table 2:** Nutritional quality of the shopping cart by allocated front-of-pack nutrition label (*N* = 2194)

	GDA (***n*** = 725)	MTL (***n*** = 734)	WL (***n*** = 735)
**Overall nutritional quality**	**Mean (SD)**	**Mean (SD)**	**Mean (SD)**
NPSC score (points/100 g/mL)^a^	1.3 (2.9)	**0.8 (3.0)**	**0.9 (3.2)**
NPSC baseline score (points/100 g/mL) ^a^	6.7 (2.9)	**6.2 (3.1)**	**6.1 (3.1)**
**Non-dairy beverages nutritional quality**			
NPSC score (points/100 g/mL)^a^	−0.9 (4.1)	**−1.5 (3.8)**	−1.1 (3.9)
NPSC baseline score (points/100 g/mL) ^a^	1.7 (2.9)	**1.3 (2.6)**	1.4 (2.7)
**Breakfast cereals nutritional quality**			
NPSC score (points/100 g/mL) ^a^	7.1 (8.8)	6.3 (9.1)	**5.9 (9.0)**
NPSC baseline score (points/100 g/mL) ^a^	13.6 (5.8)	13.0 (6.0)^b^	**12.8 (6.1)**
**Dairy nutritional quality**			
NPSC score (points/100 g/mL) ^a^	−0.0 (1.5)	−0.1 (1.3)	**0.2 (1.3)**
NPSC baseline score (points/100 g/mL) ^a^	1.7 (1.4)	1.7 (1.3)	**1.6 (1.3)**
**Ready-made foods nutritional quality**			
NPSC score (points/100 g/mL) ^a^	1.8 (4.7)	1.6 (4.9)	1.3 (4.5)
NPSC baseline score (points/100 g/mL) ^a^	7.1 (4.4)	6.7 (4.7)	6.7 (4.1)
**Salty snacks nutritional quality**			
NPSC score (points/100 g/mL) ^a^	−0.2 (5.9)	−0.7 (5.7)	**−1.0 (6.0)**
NPSC baseline score (points/100 g/mL) ^a^	16.1 (5.2)	**15.5 (5.3)**	**15.3 (5.4)**

*GDA* Guideline Daily Amount; *MTL* Multiple Traffic Light; *WL* Warning Labels; *NPSC* Nutrient Profiling Scoring Criterion

**Bolds** indicate significant differences (*p* < 0.05) with GDA. No differences were observed between MTL and WL groups. Differences were tested with unadjusted linear regression models

Sample sizes vary across food groups as follows: Non-dairy beverages: GDA = 584, MTL = 586, WL = 565; Breakfast cereals: GDA = 680, MTL = 679, WL = 683; Dairy: GDA = 708, MTL = 710, WL = 711; Ready-made foods: GDA = 585, MTL = 581, WL = 585; Salty snacks: GDA = 623, MTL = 651, WL = 618

^a^ A lower score corresponds to higher nutritional quality

^b^ Marginally significant difference (*p* < 0.06) with GDA

According to these two nutritional quality measures (NPSC score and NPSC baseline score), the WL also led to a better nutritional quality across breakfast cereals and salty snacks compared to the GDA (*p* < 0.05); a similar effect was observed for the MTL among non-dairy beverages (*p* < 0.05) (Table 2). Additionally, the NPSC score indicated that the WL led to a worse nutritional quality of dairy products (*p* < 0.05) compared to the GDA, however the opposite was observed for NPSC baseline score (*p* < 0.05). Finally, NPSC baseline score showed that the MTL led to a better nutritional quality of salty snacks compared to the GDA (*p* < 0.05).

No significant differences were observed in the NPSC score or the NPSC baseline score between the MTL and the WL, neither in the overall nutritional quality of the shopping cart, nor across food groups.

The nutritional quality of the shopping cart tended to be lower among those with the lowest household income and education levels, those not interested or a little interested in health, and those who perceived not to be knowledgeable in nutrition (*p* < 0.001) (Table 3). These effects were similar across all FOP labels. All interaction terms between label group and demographic indicators (i.e., household monthly income, education level, interest in health, and nutrition knowledge) were non-significant (all *p* > 0.05).

**Table 3:** Nutritional quality^a^ of the shopping cart across demographic and economic characteristics by allocated front-of-pack nutrition label (*N* = 2194)

	GDA	MTL	WL
	Mean (SD)	Mean (SD)	Mean (SD)
**Household monthly income (MXN pesos), mean (SD)**			
< $2699	1.9 (3.2)	1.2 (3.3)	**1.0 (3.4)**
$2700-6799	1.7 (3.0)	**1.0 (3.1)**	1.3 (3.1)
$6800-11,599	1.2 (2.7)	0.7 (3.0)	0.8 (3.3)
$11,600-34,99	0.8 (2.7)	**0.0 (2.6)**	0.4 (3.0)
> $35,000	−0.2 (2.4)	0.6 (2.6)	−0.3 (2.4)
*p-trend*	*0.000*	*0.002*	*0.003*
**Education level, n (%)**			
Secondary school and lower	2.1 (3.0)	1.7 (3.3)	1.8 (3.6)
High-school	1.5 (2.9)	**0.7 (2.9)**	1.1 (3.3)
Undergraduate	1.0 (2.8)	0.5 (2.9)	**0.4 (2.9)**
Graduate	0.9 (2.8)	−0.2 (2.7)	−0.3 (2.5)^a^
*p-trend*	*0.000*	*0.000*	*0.000*
**Interest in health, n (%)**			
Not interested or a little interested	2.3 (3.0)	1.7 (3.1)	1.8 (2.8)
Very interested	1.1 (2.8)	0.5 (3.0)	0.6 (3.2)
*p-value*	*0.000*	*0.000*	*0.000*
**Self-reported nutritional knowledge, n (%)**			
Not knowledgeable	2.3 (3.1)	1.9 (3.5)	1.9 (3.1)
A little knowledgeable	1.4 (2.7)	1.0 (2.8)	**0.8 (2.7)**
Somewhat and very knowledgeable	0.7 (2.9)	**−0.1 (2.7)**	0.3 (3.5)
*p-trend*	*0.000*	*0.000*	*0.000*

^a^ The nutritional quality of the shopping cart was evaluated using the NPSC model

**Bolds** indicate significant differences (*p* < 0.05) with GDA for a given characteristic level

*P-values* indicate differences or linear trends across levels of a given characteristic for the corresponding label

### Nutrient content of the shopping cart

Compared to the GDA, both the MTL and the WL led to lower mean nutrient content per 100 g (or 100 mL) for energy, saturated fat, sugar, and sodium (*p* < 0.05) (Table 4). Additionally, the WL led to a lower content of total fat (*p* < 0.05) (Table 4). No differences were observed between the MTL and the WL.

**Table 4:** Mean nutrient content (per 100 g/100 mL) of the shopping cart by allocated front-of-pack nutrition label (*N* = 2194)

	GDA (***n*** = 725)	MTL (***n*** = 734)	WL (***n*** = 735)
**Energy (kcal)**	202.3 (65.7)	**194.6 (68.4)**	**192.4 (66.6)**
**Total fat (g)**	9.1 (4.9)	8.9 (5.0)	**8.6 (4.7)**
**Saturated fat (g)**	2.0 (0.9)	**1.9 (0.9)**	**1.8 (1.0)**
**Sugar (g)**	9.7 (5.2)	**8.7 (5.1)**	**9.0 (5.3)**
**Sodium (mg)**	230.3 (160.2)	**214.3 (170.3)**	**204.4 (148.0)**
**Fiber (g)**	3.4 (1.6)	3.25 (1.58)	3.3 (1.7)
**Protein (g)**	7.8 (2.8)	7.6 (2.8)	7.6 (3.0)

*GDA* Guideline Daily Amount; *MTL* Multiple Traffic Light; *WL* Warning Labels

**Bolds** indicate significant differences (*p* < 0.05) with GDA. No differences were observed between MTL and WL groups. Differences were tested with unadjusted linear regression models

### Time variables and other outcomes

The MTL led to shorter time to first product selection and time spent looking at the product before deciding to buy it compared to the GDA (*p* < 0.05) (Table 5). The WL led to less total time spent shopping compared to GDA (*p* < 0.05), and marginal differences were observed between WL and GDA for the median time to first product selection (*p* = 0.060) and the median time looking at the product before deciding to buy it (*p* = 0.052) (Table 5). The proportion of participants viewing the front of the pack at least once was lower among those in the WL group compared to those in the GDA group (*p* < 0.05); meanwhile marginal differences were observed between the MTL and the GDA on this outcome (*P* = 0.057).

**Table 5:** Time variables and other outcomes by allocated front-of-pack nutrition label (*N* = 2194)

	GDA (*n* = 725)	MTL (*n* = 734)	WL (*n* = 735)
	Median (IQR)	Median (IQR)	Median (IQR)
**Time variables**			
Time to first product selection (minutes)	1.3 (0.5–2.6)	**1.1 (0.4–2.3)**	1.1 (0.4–2.2) ^a^
Time spent looking at a product before deciding to buy it (seconds)	4.0 (2.4–6.5)	**3.5 (2.3–5.8)**	3.6 (2.4–5.9) ^a^
Time spent looking at a product before deciding not to buy it (seconds)	2.5 (0–6.8)	2.6 (0–5.6)	2.9 (0–5.8)
Total time spent shopping (minutes**)**	4.7 (2.6–7.9)	4.4 (2.4–7.6)	**4.4 (2.4–7.0)**
**Other outcomes**			
Number of products in the shopping cart	20 (13–30)	20 (13–30)	20 (13–28)
Price of the products in the shopping cart (Mexican pesos)	34.7 (15.5–47.7)	35.2 (26.0–48.2)	34.7 (25.5–46.1)
Number of times the participant revised the front of the pack*	1.0 (0.3)	0.9 (0.4)	1.0 (0.5)
Proportion (%) of participants viewing the front of the pack at least once	93.8	91.1 ^a^	**89.9**

*GDA* Guidelines Daily Amount; *MTL* Multiple Traffic Light; *WL* Warning Labels

**Bolds** indicate significant differences (*p* < 0.05) with GDA. Differences in most outcomes were tested with unadjusted quantile regression models, except for the number of times the participant revised the front-of-the pack, where an unadjusted linear regression model was used. Post-hoc tests were used to test differences between MTL and WL. No differences were observed between MTL and WL groups

^a^ Marginally significant (*p* < 0.06) difference with GDA

*The mean (SD) is presented for this outcome

## Discussion

Results of this randomized experiment based on an online simulated supermarket shopping experience when consumers were prompted to make their choices with specific nutrients in mind, suggesting that the MTL and WL are more effective tools for guiding consumers towards healthier food choices than the GDA. Both the MTL and the WL improved the nutritional quality of purchases and led to reduced time when shopping in some of the studied variables compared to the GDA. Participants of low income, low education and with low nutrition knowledge had purchases with lower nutritional quality overall, across all labeling conditions.

Scarce evidence exists on the effects of FOP labels in shopping environments and situations [41]. A recent review of literature reported that only interpretive FOP labels, such as WL or the MTL, were able to modify consumers’ purchasing intentions [41]. In line with this review, our results indicate that the MTL and the WL were more effective in promoting more healthy purchasing intentions compared to the GDA, a numerical, non-interpretive FOP system. Although the effects on nutritional quality were relatively modest in magnitude, if implemented, the impact at the population level could be quite meaningful. However, based on the small differences observed between the MTL and the WL, it is possible that these two labels have similar impacts. For example, a recent study similar to ours (i.e. using an online shopping simulation) used the observed differences in nutritional content of the shopping carts between FOP labels to estimate possible changes in dietary intake and model the impact on mortality from diet-related non-communicable diseases, finding that all FOP labels tested led to reductions (i.e., 1.1–3.4%) in mortality from chronic diseases, with similar effects across most labels [42]. Future simulation modeling studies and longitudinal RCTs could shed light on the potential health benefits of MTL and WL policies.

Importantly, we prompted participants to make their choices considering specific nutrients (e.g. calories, sodium). In Latin America, studies with similar design and context have reported beneficial effects of FOP labels when inducing consumers with a health goal (e.g. preparing a healthy meal for their family) [22], but null effects when the same task is performed without inducing a health goal [21]. Indeed studies suggest that a health motivation might be needed to promote the usage of FOP labels and modify consumer behavior [26, 43, 44]. The videos used in our study were not standardized and have not been evaluated among Mexican consumers, limiting our ability to rule out any additional effects of videos above and beyond the labels’ effects. For example, the MTL and the WL videos highlighted specific chronic diseases, which were not mentioned in the GDA video. This should be explored in future studies. However, results support recommendations to combine FOP label policies with effective communication campaigns aiming to place improvements in health status and quality of life as one of the main concepts to be considered for an effective public awareness campaign aimed at promoting the use of nutritional labels [45].

Several studies have highlighted consumers’ difficulty in understanding the percentages displayed on the GDA label [8, 19, 46]. When the labelling displays descriptors or color-coding, it gives the consumer the means to interpret numeric information and reduce processing time required to interpret labels [19]. In our study, both the MTL and the WL led to improved nutritional quality and a decrease in critical nutrients that should be limited in the diet according to international agencies. While the aggregated results across all products showed similar effects for the MTL and the WL, the disaggregated results indicated differences in the performance of the WL and the MTL in guiding consumers towards healthier food choices across group categories, with more consistent results for the WL in the groups of breakfast cereals and dairy, and in the group of non-dairy beverages for the MTL. It has been suggested that although nutrient-specific systems as MTL may increase the accuracy of perceptions of nutrient contents, they may not be more effective in discouraging the selection of products with excessive content of critical ingredients as they create decisional conflicts [47]. For example, an online randomized controlled experiment comparing the effectiveness of WL against MTL among Brazilian adults reported that the MTL worsened consumer judgment for certain products (i.e. soup) [17]. In our study, products in most food categories presented a combination of colors (e.g. green, amber and red) within the same food product, which may have confused consumers or lead to prioritizing one nutrient over another. In contrast, most (11 out of 12) non-dairy beverages were labeled with a combination of two nutrients in green (e.g. sodium and fat) and one either in yellow or red (e.g. sugar), eliminating the need to prioritize between nutrients and explaining why the MTL may have been more effective in this food category. Moreover, in this food category a total of 7 beverages had no WL, with the rest having at least one WL. Therefore, the MTL may have limited ability to change consumer behavior because, in some specific cases (i.e. those presenting a combination of colors on the same product), they may not allow consumers to clearly distinguish the healthier food product [17, 48].

In our study, we were able to explore differences in the effect of the label across individual characteristics. Results suggested that nutritional quality of purchased products was consistently lower among vulnerable sub-groups (e.g. low income, low education, lower nutritional literacy), regardless labeling condition. These findings suggest that the MTL and WL are unlikely to *reduce* existent disparities in nutritional quality among vulnerable groups but are also unlikely to make existing disparities worse. Our results are in line with those indicating that nutritionally at-risk sub-groups are overall less likely to understand and use nutrition labels compared to those not at risk [28]. These sub-groups are generally more at risk of consuming a lower-quality diet, and in consequence should be a target in any FOP or other nutritional policies. Thus, closing the gap between consumers with high and low nutrition literacy may require stronger efforts, beyond simply the inclusion of a communication campaign on how to use and interpret a FOP label.

Our study also provided relevant information regarding other factors related to making food purchasing decisions. Consistent with previous studies reporting between 0.04 to 18 s required to select food products, [49, 50] participants made food choices in less than 5 s. Interestingly, the MTL and the WL helped consumers make their food choices more quickly. Results also indicated that the proportion of participants viewing the front of the pack at least once was lower among those in the WL group compared to those in the GDA group. The format and amount of information highlighted in each of the labels tested may explain these differences. For example, the GDA includes five nutrients (including calories), whereas the MTL includes three and the WL may highlight information for up to four nutrients. Also, the GDA reduces the nutritional information provided in the nutrients facts panel by highlighting the content of specific nutrients without offering any interpretation of this information, unlike the MTL and the WL which provide greater evaluation of information contained in the nutrients fact panel. Therefore, the GDA is expected to take more time to process since consumers must interpret the information provided by the label. Taken together, results indicate that both the MTL and the WL may be more visible and easier to interpret compared to the GDA, helping consumers make healthier decisions more quickly. Previous studies have also shown better objective understanding and reduced times when making food choices for these labels compared to the GDA [12].

This is the first randomized experiment in Mexico comparing the effect of different labelling systems on purchasing intentions. The greatest strength of this study is the use of a randomized experimental design, limiting the influence of confounding variables. Additionally, consistent effects were observed across study outcomes (i.e., NPSC baseline score, NPSC score and shopping times). However, several limitations should be highlighted. First, this was not a representative sample of low- to middle-income adults, limiting the representativeness of our results to populations with similar characteristics as our sample. However, the sample is large, approximates the demographic profile of the Mexican population, and includes medium to low income participants, a vulnerable population to diet-related chronic diseases. Second, we only tested three types of labels and did not include a no-label condition. However, this study provides relevant information to policy makers in Mexico and in other regions in Latin America where the GDA, the WL or the MTL have been implemented. Third, we used a limited number of food items and participants could only view the front of the pack of the products. Although we used real food products and kept any nutritional or health declarations on the frontal display of the product, we had limited ability to replicate the actual shopping experience or other potential interaction effects between the nutritional information displayed on product packages and the FOP label. Moreover, participants were prompted by videos to make their choices with specific nutrients in mind, meaning we do not know how the labels would have affected consumer behavior in the absence of these instructions. Finally, the study took place in public spaces which could have distracted participants, although we do not expect the distractions would have influenced study results since these affected participants in all of the study arms.

## Conclusion

Although GDA in Mexico was implemented since 2011, [6] the results of our study indicate that interpretative labels, such as the MTL and the WL, lead to healthier and quicker food purchase intentions compared with the GDA. However, none of the labels tested was able to produce an equitable impact among consumers with low income, education and nutrition knowledge levels. Results of this study confirm the potential of WL and MTL to foster healthier food choices in the most vulnerable groups in Mexico and underscore the need of adequately tailoring communication campaigns for this population groups.

## Supplementary information

**Additional file 1: Table S1.** Description of the videos used in the study. This table provides a general description of the three videos used to explain participants how to interpret the assigned label.

**Additional file 2: Table S2.** Nutritional quality and nutrient content of food products included in the virtual supermarket. This table provides the mean (min-max) nutritional quality, energy and nutrient (total fat, saturated fat, sugar, fiber and protein) content per 100 g/mL of the products included in the online supermarket.

## Data Availability

The datasets used and/or analyzed during the current study are available from the corresponding author on reasonable request.

## Abbreviations

FOP: Front-of-Pack
GDA: Guideline Daily Allowances
MTL: Multiple Traffic Light
WL: Warning Label
NPSC: Nutrient Profiling Scoring Criterion
