# Supplementary material for: Impact of front-of-pack nutrition labels on consumer purchasing intentions: a randomized experiment in low- and middle-income Mexican adults
Source: BMC Public Health. 2020 Apr 6;20:463. doi: 10.1186/s12889-020-08549-0 (PMC7137298; doi:10.1186/s12889-020-08549-0)
Supplement: Supplementary file 1 — Additional file 1: Table S1. Description of the videos used in the study. This table provides a general description of the three videos used to explain participants how to interpret the assigned label. [file 12889_2020_8549_MOESM1_ESM.docx]

| **FOP label** | **Produced by** | **Lenght** | **Setting** | **Main communication message** | **Audio transcription** | **Available at** |
| --- | --- | --- | --- | --- | --- | --- |
| Guideline Daily Allowances | Movimiento por una Vida Saludable (Movement for a Healthy Life), a  food industry coallition | 58 s | Animated explanation of the | Check and choose | Choose your food products wisely. Check and Choose is the front of pack nutritional labeling system that helps you know the energy, saturated fat, sugar and sodium content of foods and non-alcoholic beverages you consume. ¿How does it work? Look for it on the food packages. They show the information of 4 key nutrients. The nutrient content per portion. The percentage of the nutrient provided by the product based on the requirements of a 2000 calorie diet. Check and Choose. | <https://www.youtube.com/watch?v=6nOFexpnQgU> |
| Multiple Traffic Light | Ministry of Public Health and Ministry of Human Development Coordination | 44 s | A child with a food cart shopping at a supermarket. The child stops or keeps going depending on the color of the traffic light (label). | Choose right to live right | There are three things in foods that, if eaten in excess, may damage your engine. A lot of sugar may cause diabetes. Salt, hypertension. And fat may damage your brain and your heart. Because of that, now each processed food has a new label. Red means high content. Yellow, medium. And green, low, to be at 100%. You have the right to know what you eat. Avoid excesses. Choose right to live right. | <https://www.youtube.com/watch?v=LekdMEaETZw> |
| Warning Labels | Ministry of Health | 31 s | A family shopping at a supermarket, children shopping at a school cafeteria, adolescents skateboarding and drinking processed beverages, an older adult cooking at home. | Choose foods with fewer labels. And it’s better if they don’t have any. | With the new food law, Chile knows what is eating. Yes, because to protect childhood from overweight and other diseases, foods high in sugar, saturated fat, sodium or calories, have these warning labels. This way we take care of improving the dietary habits of everyone.  Prefer foods with fewer labels, and its better if they don’t have any. | <https://www.youtube.com/watch?v=jFjcr6uuxfg> |

Supplementary Table 1. Description of the videos used in the study.
