# Supplementary material for: Impact of front-of-pack nutrition labels on consumer purchasing intentions: a randomized experiment in low- and middle-income Mexican adults
Source: BMC Public Health. 2020 Apr 6;20:463. doi: 10.1186/s12889-020-08549-0 (PMC7137298; doi:10.1186/s12889-020-08549-0)
Supplement: Supplementary file 2 — Additional file 2: Table S2. Nutritional quality and nutrient content of food products included in the virtual supermarket. This table provides the mean (min-max) nutritional quality, energy and nutrient (total fat, saturated fat, sugar, fiber and protein) content per 100 g/mL of the products included in the online supermarket. [file 12889_2020_8549_MOESM2_ESM.docx]

**Supplementary Table 2. Nutritional quality and nutrient content of food products included in the virtual supermarket**

|  | N | Nutritional quality^a^  (NPSC score/100g) | Energy (kcal/100g) | Total fat (g/100g) | Saturated fat (g/100g) | Sugar (g/100g) | Fiber (g/100g) | Protein (g/100g) |
| --- | --- | --- | --- | --- | --- | --- | --- | --- |
| All food products | 60 | 1.9 (-8, 30) | 251 (6, 639) | 11.6 (0, 59) | 2.5 (0, 12.7) | 9.8 (0, 60) | 4.6 (0, 22.4) | 9.3 (0, 36.4) |
| Non-dairy beverages | 12 | 0.6 (-8, 27) | 79 (10, 464) | 3.3 (0, 24) | 1.9 (0, 12.5) | 7.2 (0, 35) | 0.7 (0, 4) | 2.7 (0, 26) |
| Breakfast cereals | 12 | 6.1 (-7, 21) | 381 (313, 507) | 5.5 (0, 11.5) | 1.6 (0, 7.2) | 24.3 (0, 60) | 9.6 (1.7, 17.5) | 10.3 (3.4, 16.7) |
| Dairy | 12 | 0.5 (-4, 5) | 76 (34, 183) | 2.1 (0.3, 5.1) | 1.2 (0.1, 3.2) | 7.6 (0, 17.1) | 0.2 (0, 0.8) | 6 (0.5, 36.4) |
| Ready-made foods | 12 | 2.9 (-6, 30) | 179 (6, 463) | 8.3 (0, 32.8) | 2.9 (0, 12.7) | 5 (0.6, 23.3) | 2.5 (0.2, 6.5) | 7.5 (1.2, 15.1) |
| Salty snacks | 12 | -0.8 (-8, 12) | 541 (399, 639) | 39.1 (8, 59) | 5 (1, 10) | 5.1 (0, 16.7) | 10.2 (3.4, 22.4) | 20.1 (15.2, 24) |

Numbers are means (min-max)

^a^ A lower score corresponds to higher nutritional quality
